# Snail2 induced E-cadherin suppression and metastasis in lung carcinoma facilitated by G9a and HDACs

**DOI:** 10.1080/19336918.2019.1638689

**Published:** 2019-07-11

**Authors:** Yue Hu, Yayuan Zheng, Mingrui Dai, Jiaxin Wu, Bin Yu, Haihong Zhang, Wei Kong, Hui Wu, Xianghui Yu

**Affiliations:** a National Engineering Laboratory for AIDS Vaccine, School of Life Sciences, Jilin University, Changchun, China; b Department of Gastrointestinal Surgery, China-Japan Union Hospital, Jilin University, Changchun, China; c Key Laboratory for Molecular Enzymology and Engineering, the Ministry of Education, School of Life Sciences, Jilin University, Changchun, China

**Keywords:** Snail2, G9a, HDACs, epithelial-mesenchymal transition, metastasis, lung carcinoma

## Abstract

Snail2 is a repressor of E-cadherin during carcinogenesis; however, the specific mechanisms involved in this process remain largely unknown. Here, we determined that Snail2 was highly increased during TGF-β-induced EMT process in lung cells. H3K9 methylation was up-regulated and H3K4/H3K56 acetylation were down-regulated at the E-cadherin promoter. Snail2 interacted with G9a and HDACs to exert suppression of E-cadherin transcription. Overexpression of Snail2 enhanced the migration and invasion ability, whereas G9a and HDACs inhibition significantly reversed this effect. Our study demonstrated the importance of G9a- and HDACs-mediated regulation during Snail2-induced E-cadherin repression and metastasis during LC progression.

## Introduction

Among all carcinomas, lung carcinoma (LC) is the most common cause of cancer-related death [1]. Therapeutic resistance and tumor metastasis are the most common causes of lung cancer-related death. Despite recent progress in early detection and therapeutic efficacy, but is hampered because of their high recurrence rates and the development of metastasis [2]. Metastasis is initiated by a process in which tumor cells disseminate and gain invasive ability, a step referred to as epithelial-to-mesenchymal transition (EMT) [3].

EMT is an evolutionary conserved trans-differentiation process proposed to play a crucial role in cancer metastasis [4]. During EMT, activated cells gain stem cell-like features, which provide a distinct advantage for tumor progression and metastasis [5,6]. Upon the completion of EMT, the expression of epithelial markers like E-cadherin is suppressed, whereas mesenchymal markers such as vimentin and fibronectin are upregulated [7]. The loss of E-cadherin expression is heavily involved in EMT, and E-cadherin is therefore emerging as one regulator of the epithelial phenotype. Genes encoding various transcription factors including Snail (SNAI1), Slug (SNAI2), TWIST1, and ZEB1 become activated following the extracellular stimuli and carry out the downstream nuclear regulation of EMT [8].

Snail2 (also referred to as Slug) belong to the Snail superfamily of zinc finger transcriptional repressors that participate in developmental EMT [9]. Snail2 has been reported to induce EMT in SW480 by suppressing E-cadherin expression [7]. Reduced E-cadherin expression has been shown to be associated with poorly differentiated and metastatic NSCLC. In NSCLC, knockdown of SNHG12 suppresses tumor metastasis and epithelial-mesenchymal transition via the Snail2/ZEB2 signaling pathway by targeting miR-218 [10]. It has been reported that NatD promotes lung cancer progression by preventing histone H4 serine phosphorylation to activate Snail2 expression [11]. However, few studies have investigated the relationship between Snail2 and E-cadherin during EMT in lung carcinoma, and its implications in tumor progression.

G9a, a histone H3 lysine 9 (H3K9) methyltransferase [12,13], has been implicated as an important oncogenic driver in multiple cancers [14–16]. One previous report showed that G9a can interact with Snail1 and DMNTs to participant in Snail-mediated E-cadherin repression in breast cancer [17]. HDACs, which are deacetylases, can facilitate transcriptional repression and the formation of heterochromatin [18]. Some studies have demonstrated that in general, aberrant histone deacetylation modifications are related to cancer [7]. It has been reported that HDAC1, 2, and 3 were highly expressed and excessively activated in prostate cancer [19]. Moreover, there is a growing body of evidence showing that the expression of class I HDACs was increased in ovarian carcinomas [20–22].

In the present study, we investigated the epigenetic program of EMT in LC by focusing on the transcriptional regulation of E-cadherin. We found that Snail2 was remarkably increased in primary LC and highly expressed in TGF-β-induced EMT in lung cells. Furthermore, we demonstrated that levels of H3K9methylation, H3K4 acetylation and H3K56 acetylation were changed in the promoter region of E-cadherin. In addition, G9a and HDACs acted together with Snail2 at the E-cadherin promoter to suppress transcription. Snail2 was found to interact with G9a and HDACs, indicating that they might form a functional complex to repress E-cadherin expression. We validated that the overexpression of Snail2 significantly enhanced migration and metastatic capacity at the cellular level and in a mouse model. Thus, our studies indicate that Snail2 can interact with G9a and HDACs to mediate the repression of E-cadherin, thereby promoting the migration and invasion in human malignant carcinoma.

## Results

### Snail2 is highly expressed in LC

**Figure 1.**
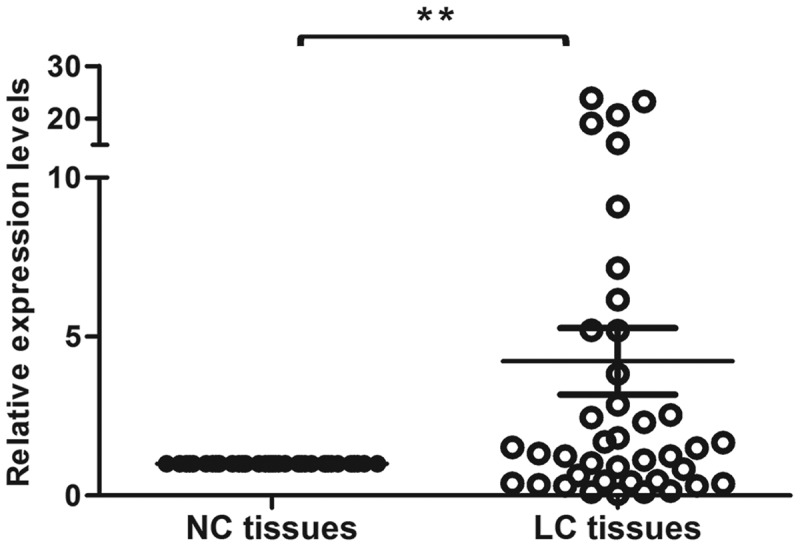
Expression levels of Snail2 in lung carcinoma. The expression levels of Snail2 in 40 paired lung carcinoma (LC) tissues and NC tissues were quantified by qRT-PCR. Data represent the mean ± SD; * *P* ≤ 0.05, **P ≤ 0.01.

To explore the potential role of Snaill2 in lung carcinoma, we examined the expression level of Snail2 in paired cancerous and corresponding adjacent non-cancerous tissues (Figure 1). We found that the mRNA level of Snail2 was remarkably up-regulated in LC tissues. Thus, our results suggest that Snail2 might play different functions in LC and that Snail2 might be involved in the tumorigenesis and metastasis of LC.

### Snail2 participates in TGF-β-induced EMT in lung cells and LC cells

**Figure 2.**
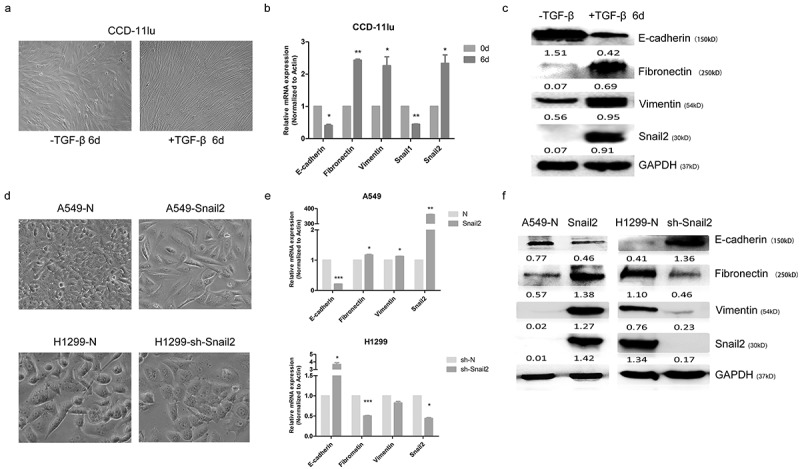
Expression profiles of epithelial and mesenchymal markers during TGF-β-induced EMT in lung cell and the change of cell morphology and expression levels of EMT markers after Snail2 modulation in LC. (A) The lung cell line CCD-11lu was treated with TGF-β1 (10 ng/ml) for 6 days; cell morphological changes associated with EMT are shown as phase contrast images. Phase contrast images were taken at 10× magnification. (B) The CCD-11lu cell line was treated with TGF-β1 (10 ng/ml) for the indicated time periods. mRNA levels of genes encoding E-cadherin, N-cadherin, fibronectin, vimentin, Snail1 and Snail2 in these cells were analyzed by qRT-PCR and shown as ‘relative mRNA levels’. Data represent the mean ± SD. *P ≤ 0.05; **P ≤ 0.01; ***P ≤ 0.001. (C) Protein levels of E-cadherin, fibronectin, vimentin, and Snail2 in the cell line was analyzed by western blotting. 40 μg proteins from each sample was resolved by SDS-polyacrylamide gel electrophoresis (PAGE)/were loaded and separated by SDS-polyacrylamide gel electrophoresis (PAGE). (D) Cell morphology A549-Snail2, A549-N cells, H1299-sh-Snail2 and H1299-N cells, as shown by phase contrast images. Phase contrast images were taken at 20× magnification. (E) mRNA levels of genes encoding an epithelial marker (E-cadherin) and mesenchymal markers (fibronectin and vimentin) were analyzed by qRT-PCR in these cells lines. (F) Protein levels of the epithelial marker (E-cadherin) and mesenchymal marker (fibronectin) were analyzed by western blotting in these cell lines. 30 μg proteins from each A549 cell lines samples and 40 μg proteins from each H1299 cell lines samples were resolved by SDS-polyacrylamide gel electrophoresis (PAGE)/were loaded and separated by SDS-polyacrylamide gel electrophoresis (PAGE).

We next confirmed the function of Snail2 by investigating TGF-β-induced EMT in the lung cell line CCD-11Lu. We found that treatment with 10 ng/ml of TGF-β1 for 6 days induced CCD-11Lu cells to undergo EMT. As expected, TGF-β treatment resulted in the acquisition of fibroblastic mesenchymal morphology in CCD-11lu cell line (Figure 2A), which was accompanied by the downregulation of epithelial markers including E-cadherin and the upregulation of mesenchymal markers including Fibronectin and Vimentin at the RNA level (Figure 2B) and protein level (Figure 2C) in the model cell line. Our results showed that Snail2 along with the downregulation of E-cadherin, in CCD-11Lu cells, indicating that TGF-β-induced EMT in lung cells was associated with Snail2 expression.

To validate the function of Snail2 in LC cells, we further established stable Snail2-overexpressing cell lines using poorly metastatic A549 cells (designated as A549-Snail2) and also knocked down the expression level of Snail2 in the highly metastatic H1299 cells (designated as H1299-sh-Snail2). We found that A549-Snail2 cell line exhibited a fibroblastic, mesenchymal morphology, which was not apparent in respective control cells (that had an epithelial phenotype) (Figure 2D). Meanwhile, Snail2 knockdown in H1299 cell line resulted in the acquisition of epithelial morphology (Figure 2D). This observation was also confirmed by qPCR and western blot analyses of the expression levels of an epithelial marker (E-cadherin) and mesenchymal markers (fibronectin and vimentin). We found that Snail2 overexpression in A549 cell line significantly decreased the expression of E-cadherin, and increased the expression of fibronectin and vimentin (Figure2E,F). In contrast, silencing Snail2 in H1299 cells resulted in the upregulation of E-cadherin and the downregulation of fibronectin and vimentin (Figure 2E,F). These findings supported that Snail2 could induce EMT in LC cell lines.

### G9a and HDACs are required for the snail2-mediated repression of the E-cadherin through H3K9 methylation and H3K4 and H3K56 deacetylation in LC

**Figure 3.**
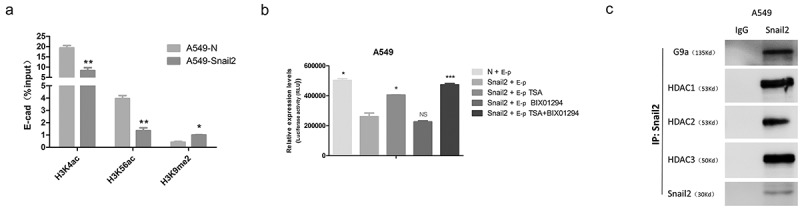
Epigenetic modifications at the promoter of E-cadherin in Snail2-overexpressing cell line and the function of Snail2, G9a and histone deacetylases (HDACs) on the activity of E-cadherin promoter. (A) Levels of H3K9 dimethylation (H3K9me2), H3K4 acetylation (H3K4ac) and H3K56 acetylation (H3K56ac) at the promoter of E-cadherin in the A549-Snail2 cell lines, as well as their respective control cell lines, were assessed by CHIP. ‘Percentage of input’ indicates the ratio of the DNA fragment of each promoter region bound by H3K9me2, H3K4ac and H3K56ac, to the total amount of input DNA without H3K9me2-, H3K4ac-, and H3K56ac-, specific antibody pull-down. *P ≤ 0.05; **P ≤ 0.01; ***P ≤ 0.001, based on the Student’s t test. All results are from three independent experiments. Data represent the mean ± SD. Luciferase reporter assays were carried out in 293T cells (B) Luciferase reporter assays were carried out in A549-Snail2 cells that were transfected with a plasmid harboring the E-cadherin promoter and treated with Bix-01294 (5 μM) or TSA (1 μM). Luciferase activity was assayed 24 h later and normalized to that of *Renilla* (pRL-SV40), which served as an internal control. Each data point represents the mean ± SD. Experiments were performed twice in triplicate. (C) Co-immunoprecipitation assays performed in A549-Snail2. Cell extracts were immunoprecipitated separately with Snail2 antibodies, and bound endogenous G9a and HDACs were examined by western blotting.

Chromatin modifications including histone methylation and deacetylation play crucial roles in suppressing gene expression. To determine whether Snail2 can epigenetically regulate the expression of E-cadherin, we examined levels of repressive histone modifications at the E-cadherin promoter using ChIP assays. As one of the most important repressive modifications, the H3K9me2 euchromatic histone methylation marker was remarkably up-regulated at the E-cadherin promoter in A549-Snail2 cells (Figure 3A). Furthermore, a significantly reduction of H3K4ac and H3K56ac markers at the E-cadherin promoter was detected in A549-Snail2 cell line (Figure 3A).

To investigate the cofactors involved in Snail2-mediated repression of E-cadherin, a truncated E-cadherin promoter region (−108 bp to +126 bp) was sub-cloned into the pGL3-basic vector (designated as E-p). We determined if G9a (the histone H3K9 methyltransferase) and HDACs (the histone H3K4 and H3K56 deacetylation) cooperate to repress E-cadherin in LC. In the A549 cell line, inhibition of G9a or HDACs alone slightly up-regulated activity of the E-cadherin promoter compared to that in Snail2-overexpressing cells, whereas inhibition of both simultaneously remarkably rescued the Snail2-mediated repression of E-cadherin promoter (Figure 3B).

The involvement of Snail2, G9a, and HDACs in the transcriptional inhibition of E-cadherin suggests that these molecules might interact with each other. We performed Co-IP experiment in A549-Snail2 cell line and found that endogenous G9a, HDAC1, HDAC2, and HDAC3 were selectively co-immunoprecipitated using specific antibodies to Snail2, demonstrating the existing of Snail2-G9a-HDACs complex in vivo (Figure 3C). Hence, the observation demonstrated that Snail2 can interact with G9a, HDAC1, HDAC2 and HDAC3 to form a multi-complex that participates in the transcriptional inhibition of E-cadherin. Taken together, our results demonstrated that G9a and HDACs are required for Snail2-mediated repression of E-cadherin by mediating H3K9 methylation, as well as H3K4 and H3K56 deacetylation, in the promoter region in LC.

### G9a and HDACs mediate the migration, invasion-, and metastasis-promoting properties of snail2 in LC cells

**Figure 4.**
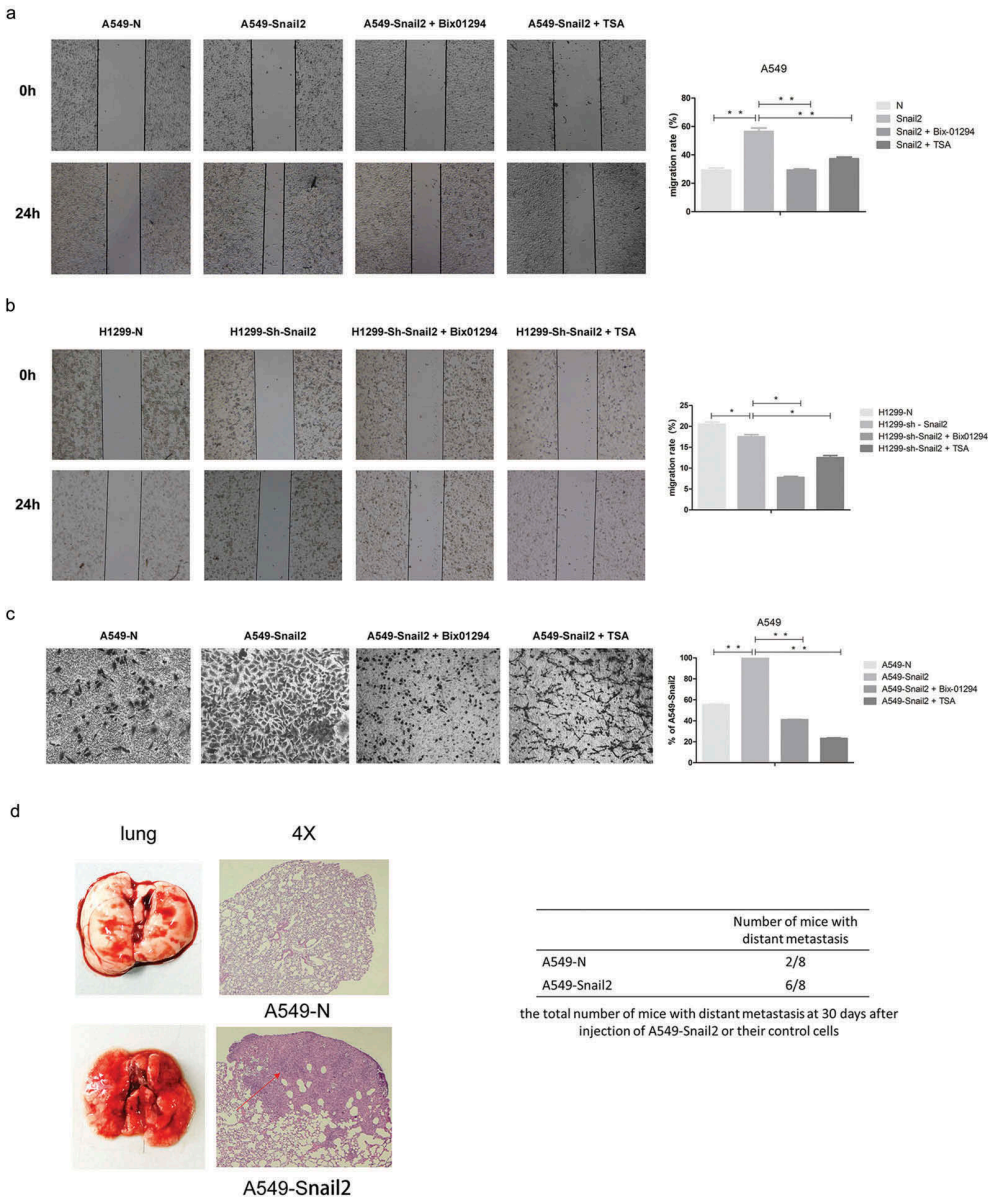
Functions of Snail2, G9a and HDACs facilitate on the migration, invasion, and metastasis. (A) Migratory ability of A549-N cells, A549-Snail2 cells, and A549-Snail2 cells treated with Bix-01294, and A549-Snail2 cells treated with TSA, as analyzed by wound healing assays. (B) Migratory ability of H1299-N cells, H1299-sh-Snail2 cells, H1299-sh-Snail2 cells treated with Bix-01294, and H1299-sh-Snail2 cells treated with TSA, as analyzed by wound healing assays. (C) Invasiveness of A549-N cells, A549-Snail2 cells, A549-Snail2 cells treated with Bix-01294, and A549-Snail2 cells treated with TSA, as analyzed by transwell invasion assays. Statistical analysis of invasion is shown in the bar graph (mean ± SD from three independent experiments), and a representative experiment is shown in the right panel. Phase contrast images were taken at 4 × magnification. (D) A549-Snail2 cells and control cells were subcutaneously injected into nude mice. After 30 days, mice were sacrificed and the lung was dissected. Lung metastatic nodules were examined macroscopically or paraffin-embedded sections were subjected to H&E. Arrowheads indicate liver metastases. Phase contrast images were taken at 4 × magnification. (E) Total number of mice with distant metastasis 30 days after injection of A549-Snail2 or respective control cells.

Because G9a and HDACs are required for the Snail2-mediated repression of E-cadherin in LC cells, we next investigated their cooperative functions with respect to the invasion and metastasis of LC. Compared to those in control cells, the overexpression of Snail2 in A549 cells significantly increased cell migratory and invasive abilities (Figure 4A,C). In contrast, the downregulation of Snail2 in H1299 cells remarkably reduced the rate of migration (Figure 4B), demonstrating that this factor can enhance the migratory and invasive capacities of LC cells in vitro. We then treated these cell lines with the G9a inhibitor Bix-01294 and the HDAC inhibitor TSA and further compared migration and invasion to those in untreated cells. Our results showed that G9a inhibition significantly reduced the migratory and invasive capacity of A549-Snail2 cells (Figure 4A,C). Meanwhile, the functional suppression of HDACs activity also markedly reduced the rate of migration and invasion in A549-Snail2 cells (Figure 4A,C). Thus, these data indicated that G9a and HDACs cooperate with Snail2 to enhance the migration and invasion of LC cells.

Furthermore, we extended our findings to a xenograft model of metastasis, in which LC cells are subcutaneously injected into mice to generate pulmonary metastases. A549-Snail2 and respective control cells were administered to nude mice to investigate the tumorigenic and metastatic potential of these cells. We noticed that mice administered A549-Snail2 cells had an increased number of lung metastasis, as compared to that observed in A549-N-injected mice (Figure 4D). In addition, we used an experimental metastasis model, in which tumor cells were directly injected into the tail veil of nude mice, to confirm these findings. Results showed that the overexpression of Snail2 in LC cells remarkably increased distant metastasis in mice, demonstrating that this factor can promote LC migration and invasion in vivo (Figure 4E). Together, our results confirmed that G9a and HDACs facilitate the Snail2-mediated migration, invasion, and metastasis of LC cells.

## Discussion

Most of previous studies focused on the developing embryos regulation ability of Snail2, whereas other roles of Snail2 in cancer seldom investigated. Recently, Snail2 has been implicated in different types of malignancies [9]. In our preceding study, we found that Snail2 could suppress E-cadherin expression by recruiting HDAC6 and PRC2 in colorectal cancer [7]. The present study suggests that Snail2 also function as an activator for TGF-β induced repression of EMT in lung carcinoma. But, unlike colorectal cancer, Snail2 induced E-cadherin repression and metastasis via G9a and HDACs.

In previous studies, Snail family proteins were shown to participate in EMT in diverse carcinomas [23,24]. It has been reported that Snail1 activation and consequent repression of E-cadherin may depend on AKT-mediated NF-κB activation [25]. In colorectal carcinomas, Snail1 could combine with the competitive displacement of ASCL2 and epigenetic mechanisms to rapidly silence the EPHB3 tumor suppressor [26]. Moreover, Snail1 could mediate E-cadherin repression by the recruitment of Sin3A/HDAC1/HDAC2 complex [27]. However, the mechanism through which Snail2 controls the silence of E-cadherin in lung carcinoma remains undefined. In this study, we first confirmed that Snail2 was significantly upregulated in LC tissues. Consistent with this finding, Snail2 was remarkably increased during TGF-β-induced EMT in lung cells. Overexpression of Snail2 induced EMT in LC cells, whereas knockdown of this factor resulted in the reversion of LC cells from a fibroblast-like morphology to an epithelial phenotype. Thus, all those experiments fully proved that Snail2 is indispensable to EMT process in lung carcinoma.

Epigenetic regulation has been suggested to be involved in EMT process in several malignant carcinomas. A previous study showed that UTX was found to inhibit EMT-induced breast cancer stem cell properties, via the epigenetic repression of EMT-associated genes, in cooperation with LSD1 and HDAC1 [28]. It has been reported that HDAC1, 2, and 3 were highly expressed and excessively activated in prostate cancer [19]. The EZH2-H3K27me3-DNMT1 complex orchestrated epigenetic silencing of the wwc1 gene, a Hippo/YAP pathway upstream effector, in breast cancer epithelial cells [29]. In our study, we demonstrated that Snail2 interacted with G9a and HDACs to form a multi-protein complex at the promoter of E-cadherin. Further, G9a and HDACs were found to catalyze H3K9 di-methylation and H3K56/H3K4 deacetylation at the E-cadherin promoter, and finally trigger the suppression of E-cadherin and EMT process in LC cells. Moreover, the overexpression of Snail2 remarkably enhanced migration, invasion, and metastasis in LC cell line. However, the G9a and HDACs inhibition significantly reversed these effects.

Hence, all those data collectively indicated that Snail2 could promote EMT process and metastasis of Lung cancer and that G9a and HDACs are both crucial for the suppression of E-cadherin and metastasis in LC cells. Consequently, Snail2/G9a/HDACs axis might represent a therapeutic target for the treatment of aggressive and metastatic lung tumors.

## Materials & methods

### Cell culture and transfection

A549 cell lines were grown in DMEM supplemented with 10% FBS and H1299 cell lines were grown in RPMI-1640 plus 10% FBS in a humidified atmosphere and 5% CO_2_, at 37°C. Cells were purchased from the Cell Bank of Type Culture Collection of the Chinese Academy of Sciences, Shanghai Institute of Cell Biology, Chinese Academy of Sciences. A549 cells were infected with a lentivirus expressing EGFP and Snail2 or their control lentivirus (Hanbio) at an MOI of 10. H1299 cells were infected with a lentivirus expressing EGFP and Sh-Snail2 or their control lentivirus (Hanbio, China) at an MOI of 20. Stable cell lines were selected using puromycin (1 mg/mL, Sigma). sh-RNA (Snail2) sequence 5ʹ-GATGCATATTCGGACCCACACATTA-3ʹ.

### Quantitative real-time polymerase chain reaction

Total RNA from tissue samples was isolated with TRIzol (Invitrogen). Quantitative real-time PCR was performed in the CFX96 Real-Time PCR Detection System (Bio-Rad) using TransStart Top Green qPCR SuperMix (Transgen) according to the manufacturer’s instructions.Quantitative real-time polymerase chain reaction data was analyzed using the comparative Ct method, and the expression of target genes were normalized to that of β-actin.

### Western blotting and antibodies

Protein samples were mixed with 4× SDS loading buffer. Samples were boiled for 15 min at 98°C, and proteins were separated by Biofuraw™ Precast Gel (Tanon) and transferred to NC membranes, and then, the following primary antibodies and dilutions were used: Snail2 monoclonal antibody (1:1000, c-166,467, Santa Cruz,), E-cadherin polyclonal antibody, vimentin polyclonal antibody, fibronectin polyclonal antibody, and GAPDH polyclonal antibody (1:1000, 874–1-AP, 15,613–1-AP, 10,366–1-AP, 60,004–1-AP, Proteintech). Secondary antibodies were anti-rabbit or anti-mouse HRP-conjugated IgG (1:2000, SA00001-4, Proteintech). Membranes were incubated with 1 mL ECL western blotting substrate (Promega) for 1 min at room temperature and then exposed to x-ray film.

### Chromatin immunoprecipitation (chip) assays

Chromatin immunoprecipitation assays were performed according to the protocol described previously [30]. Cells (1 × 10^7^) grown to 80% of confluence were used for each ChIP. H3K9me2 (C15200154, 5 μg/ChIP), H3K4ac (C15410322, 5 μg/ChIP), and H3K56ac (C15410213, 5 μg/ChIP) antibodies were purchased from diagenode.The primers for the E-cadherin promoter was: 5′-GCCCTTTCTGATCCCAGGTC-3′ and 5′-TAGCCTGGAGTTGCTAGGGT-3′.

### Luciferase assay

For luciferase assays, the Dual-Luciferase Reporter Assay System (Promega) was used as previously described [31]. Briefly, these cells were co-transfected with an E-cadherin-promoter containing luciferase construct together with a plasmid expressing Renilla luciferase (pGL3-Bisic, Promega). Firefly luciferase activity was normalized to Renilla luciferase activity which was a control for transfection efficiency. All experiments were performed 3 times in triplicate.

### Wound healing assays

Wound healing assays were performed as described previously [32,33]. Cells were seeded in 6-well plates (1 × 10^6^ cells/well) and cultured at 37°C. After 24 h, a pipette tip was used to create a wound in the cell monolayer. The cells were then cultured in RPMI-1640 or DMEM supplemented with 2% FBS at 37°C. Inhibitors, Bix-01294 (S8006, Selleck) and TSA (S1045, Selleck) if used, were added to the complete medium. The width of the wound was measured under a microscope (Nikon, DS-U2) 24 h after the scratch.

### Invasion assays

Invasion assays were performed as described previously [34]. Boyden chambers were coated with Matrigel (BD Biosciences). According to the manufacturer’s protocol, cells (5 × 10^3^) in serum-free medium with inhibitors were seeded on Matrigel in the upper chamber, and the bottom chamber was ﬁlled with culture medium containing 10% FBS. Cells that invaded through the Matrigel-coated membrane after 24 h were ﬁxed with paraformaldehyde, and stained with crystal violet. The fold change in invasion was calculated by dividing the number of cells in A549-Snail2 and H1299-sh-Snail2 by the number of cells in the control cells. All experiments were conducted at least twice in triplicate.

### Experimental lung metastasis models

Female BALB/c nude mice (6–8 weeks old) were purchased from Charles River. All animal studies were conducted in accordance with legal and institutional guidelines. The procedures were approved by the Ethical Committee of Care and Use of Laboratory Animals at Jilin University. Mice were injected with A549-Snail2 cells (5 × 10^6^ cells/mouse) and control cells via subcutaneous injections (8mice/group). After 30 days, mice were sacrificed. Visible lung metastatic nodules were examined macroscopically or embedded in paraffin, sectioned, and subjected to H&E.

### Statistical analysis

The GraphPad Prism software for Windows (GraphPad Software) was used for all statistical analyses. The results are expressed as mean values ± SD. Significant differences between two groups were assessed using paired two-tailed Student’s t-tests. A *P*-value < 0.05 was considered statistically significant.

## Figures and Tables

**Table:** 

LC	lung carcinoma
EMT	epithelial-mesenchymal transition
HDAC	histone deacetylase
TGF	transforming growth factor
